# An examination of Thailand’s health care system and strategies during the management of the COVID-19 pandemic

**DOI:** 10.7189/jogh.11.03002

**Published:** 2021-01-13

**Authors:** Alwin Issac, Rakesh Vadakkethil Radhakrishnan, VR Vijay, Shine Stephen, Nadiya Krishnan, Jaison Jacob, Sam Jose, SM Azhar, Anoop S Nair

**Affiliations:** College of Nursing, All India Institute of Medical Sciences (AIIMS), Bhubaneswar, India

In the Global Health Security (GHS) index ranking, Thailand occupies the sixth position and is the only non-high-income country that was able to secure a position among the top ten. The GHS index ranks all nations according to their health security, readiness against epidemics, and related capabilities [1]. Thailand consistently stands among top ranked nations in the Global COVID-19 recovery Index (GCI). GCI evaluates key recovery parameters reported daily and gives an overview of the nations’ fight against the Coronavirus disease-2019 (COVID-19). This allows other nations to identify and adopt the most suitable measures that aid in the fight against containment of virus [2]. The success story of Thailand’s health care system is not a single day miracle, instead it is the result of their consistently executed strategies over the past many decades. In this viewpoint, the authors examined the strategies behind the success of Thailand in containing the COVID-19 pandemic.

Thailand makes up 0.9% of the total world population and was the first nation that recorded COVID-19 cases outside China. Bangkok city, with a population density of 17 425 people per-square mile, was predicted to be most affected by COVID-19 based on the influx of travelers from the affected cities of Mainland China [3]. Throughout the pandemic, Thailand has demonstrated several successful elements in the different phases of preparedness and response framework.

## PREPAREDNESS

Enhancement of health along with education was given priority in the government’s National Health Development Plan (1960-75). In response to this, the district health system development scheme was established in 1977 to ensure the establishment of district hospitals and health centers that would have sufficient health professionals catering to entire districts. With their enduring investment in health care organization and qualified health care personnels, Thailand attained universal health care coverage in 2002 [4]. Thailand executed their pandemic preparedness strategies in accordance with their national pandemic influenza preparedness plan [5]. People of Thailand are used to wearing masks against air pollutants and is the lonesome Asian country that displayed “universal precaution” in combating COVID-19. The Ministry of Public Health introduced “Big cleaning week”, to create awareness among the people regarding personal hygiene practices at workplace, residence, and public places. Biometric fever screening was installed at the airports after being integrated into existing biometric border control system well before Thailand recorded their index COVID-19 case. Except for ferrying Thai citizens from abroad, international flights were banned from entry. Passengers were expected to show medical documents before boarding their flights to Thailand and later at immigration checkpoints, and they were to seek quarantine for a fortnight in a government managed setting [6].

**Figure:**
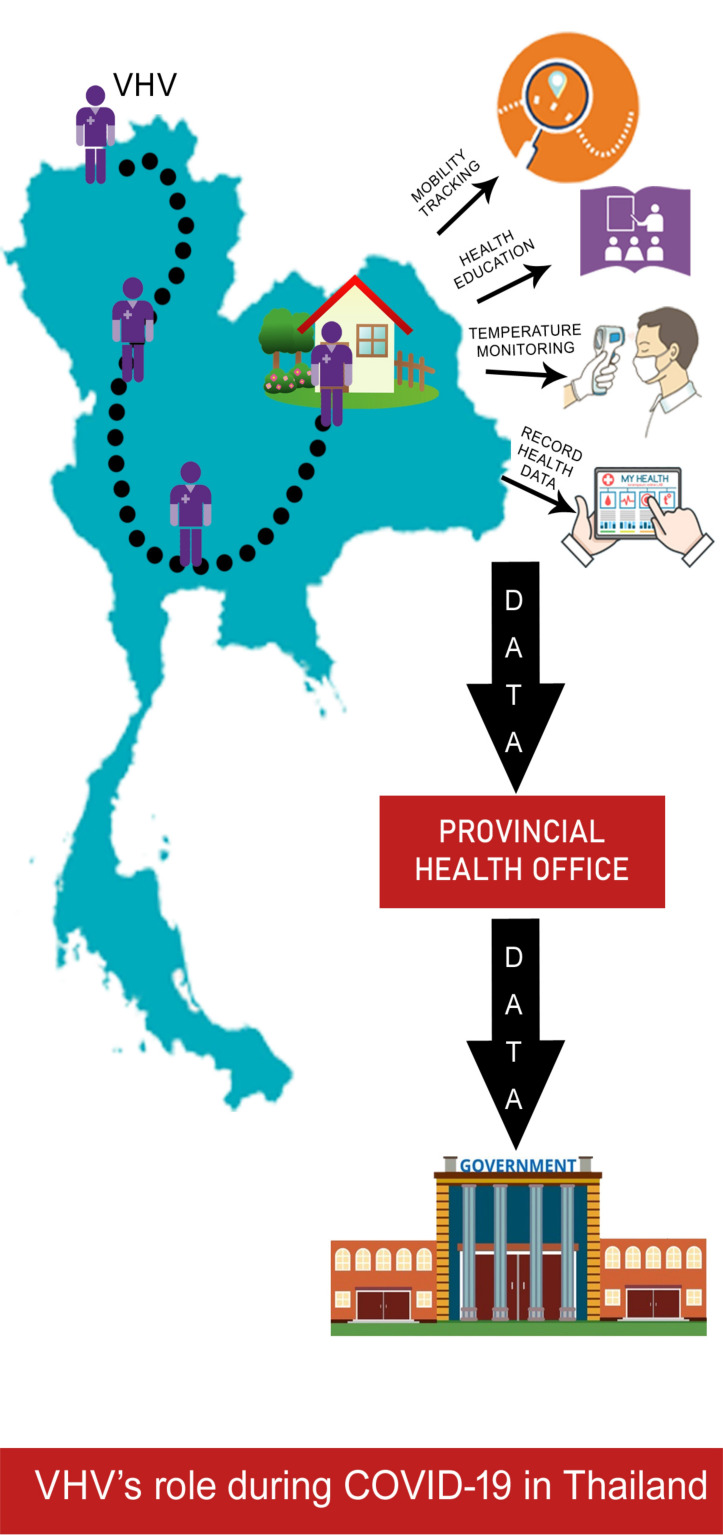
Photo: Village Health Volunteers (VHV’s) role during COVID-19 pandemic in Thailand (from the author’s own collection, used with permission).

## SURVEILLANCE

On January 13, 2020, a Chinese tourist to Bangkok from Wuhan tested positive for COVID-19. Anticipating economic constraints, testing was optimized to all potential cases, and hence contact tracing was much strengthened. A self-reporting online tool and a tracking application “Thai Chana” was adopted to facilitate contact tracing. A quick response (QR) code scanning was mandatory for every person entering a shop or restaurant. This later facilitated contact tracing [7]. Health warning cards were issued to travelers arriving in Thailand, which had to be shown to doctors in case they experienced any COVID-19 symptoms within 14 days of arrival in Thailand.

## CONTAINMENT

Meticulously executed Risk Communication and Community Engagement (RCCE), drafted by WHO were crucial in informing and guiding the public during a pandemic, and proved to be indispensable to successfully respond to health emergencies. RCCE minimizes the knowledge gap between originators of information and the public; thereby ensuring that the public is made aware of the risks surrounding the epidemic and understands their role and responsibility in slowing the spread of the virus until a vaccine or treatments become readily available. The Centre for COVID-19 Situation Administration (CCSA) was set-up to officially announce the COVID-19 situation, and they centralized the entire anti-virus campaign in the country [6,8]. On January 23, the government stopped all non-essential travel to China and imposed a curfew. Screening systems and alcohol based sanitizers were installed at tourist destinations, travelers were advised to wear a mask, public transportation was thoroughly disinfected, and export of surgical masks was curtailed [9]. A nation-wide emergency was declared on March 26 until August 31. All non-essential businesses were shut down along with schools, entertainment venues and public gatherings by mid-March. Festivities in villages were banned, the markets thoroughly cleansed, and stringent physical distancing measures were practiced even at funerals [9].

## RESPONSE

**Figure 1:**
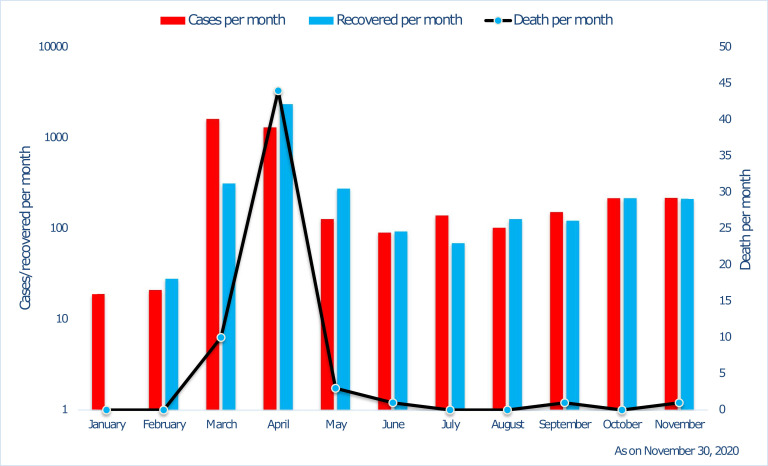
Timeline since the first COVID-19 case in Thailand.

Thailand’s health care workforce density is above World Health Organization (WHO) benchmark, which in-turn helped in proper management of COVID-19 crisis [10]. As an innovative treatment regimen, patients with severe symptoms of COVID-19 were treated with anti-influenza (Oseltamivir) and anti-retroviral (Lopinavir and Ritonavir) drugs [11]. The medical emergency support system ranged from the first responder (FR) level to the advanced life support (ALS) level through basic life support (BLS). In an emergency, Thailand has a unique approach to providing first responders. Volunteers who manage 65 percent of emergency cases are sent out, and full-fledged ambulances with professional support are delegated if deemed necessary. Many volunteers perform the FR level and are associated with foundations and local administrative bodies [8]. Though the first COVID-19 case outside China was reported in Thailand, which has a very large population of 69 million; the country was able to keep the cases and mortality in check within a few months (**Figure 1**).

Thailand’s effective COVID-19 management is mainly due to the role played by their community health workers, designated as “village health volunteers” (VHVs). They supervised individuals’ mobility, carried out household visits for temperature monitoring, communicated health messages pertaining to coronavirus disease and how to prevent it, recorded household health data, and conveyed this data to the provincial health office and thereafter to the central government. VHVs were set up in 1977 as part of government efforts to cater to rural communities. Soon after the end of first wave of the COVID-19 pandemic, villagers who had originally employed away from the village returned to their residence and a door-to-door education on COVID was carried out by VHVs. They gathered information on villagers’ travel history and health status from the village headman daily. Each VHV caters to 10-15 families and most of them are women. With basic health training, they provide rudimentary care and initial diagnoses in areas that are often far away from a clinic or hospital, and they link the community to the organized health system. Apart from conducting home visits and educating public, they monitor people undergoing 14-day isolation and issue permits following the period. Violators are arrested, and appropriate reformatory measures are instituted [8].

While the government relied on an emergency decree, in Thailand we saw a response driven by public health employees emphasizing on preventive measures. Direct daily fact-based communication between professionals and the public resulted in better awareness, and subsequently translated into solid understanding and co-operation. Traditionally observed norms of personal hygiene, voluntary abidance of civilians, voluntary aid of hundreds of thousands of grass-root public health activists, and collaboration between public health authorities and civil society; paved the way for a successful management and containment of COVID-19. The country has gone back to business as usual, though precautionary measures of using mask and social distancing are practiced. Thailand reiterates the significance of unceasing investments in primary health care to develop a robust health system.
